# Cascade Amplified Plasmonics Molecular Biosensor for Sensitive Detection of Disease Biomarkers

**DOI:** 10.3390/bios13080774

**Published:** 2023-07-31

**Authors:** Hsin-Neng Wang, Tuan Vo-Dinh

**Affiliations:** 1 Fitzpatrick Institute for Photonics, Duke University, Durham, NC 27708, USA; hsinneng.wang@duke.edu; 2 Department of Biomedical Engineering, Duke University, Durham, NC 27708, USA; 3 Department of Chemistry, Duke University, Durham, NC 27708, USA

**Keywords:** surface-enhanced Raman scattering, SERS, plasmonics, nanobiosensors, nanoprobes, microRNA detection, nucleic acid detection, signal amplification, molecular diagnostics

## Abstract

Recent advances in molecular technologies have provided various assay strategies for monitoring biomarkers, such as miRNAs for early detection of various diseases and cancers. However, there is still an urgent unmet need to develop practical and accurate miRNA analytical tools that could facilitate the incorporation of miRNA biomarkers into clinical practice and management. In this study, we demonstrate the feasibility of using a cascade amplification method, referred to as the “Cascade Amplification by Recycling Trigger Probe” (CARTP) strategy, to improve the detection sensitivity of the inverse Molecular Sentinel (iMS) nanobiosensor. The iMS nanobiosensor developed in our laboratory is a unique homogeneous multiplex bioassay technique based on surface-enhanced Raman scattering (SERS) detection, and was used to successfully detect miRNAs from clinical samples. The CARTP strategy based on the toehold-mediated strand displacement reaction is triggered by a linear DNA strand, called the “Recycling Trigger Probe” (RTP) strand, to amplify the iMS SERS signal. Herein, by using the CARTP strategy, we show a significantly improved detection sensitivity with the limit of detection (LOD) of 45 fM, which is 100-fold more sensitive than the non-amplified iMS assay used in our previous report. We envision that the further development and optimization of this strategy ultimately will allow multiplexed detection of miRNA biomarkers with ultra-high sensitivity for clinical translation and application.

## 1. Introduction

In recent years, it has been demonstrated that microRNAs (miRNAs) are essential regulators in various biological processes, including oncogenesis and cancer progression [1,2,3,4,5,6,7]. These small noncoding endogenous RNAs can function as oncogenes and tumor suppressors [8,9]. For example, overexpressed miRNAs can downregulate the expression of tumor suppressor genes by binding to the 3′ untranslated regions (UTRs) of their target mRNAs leading to tumorigenesis. Recent studies have shown that miRNA expression is dysregulated in many diseases, including cancer, infectious diseases, cardiovascular diseases, etc. [10,11,12]. In particular, miR-21, one of the most intensively studied miRNAs, has been described as an oncomiR, which is overexpressed in many cancers, including the aggressive triple-negative breast cancer, esophageal adenocarcinoma and colorectal cancer [13,14,15,16,17,18,19]. Moreover, it has been shown that miR-21, together with other miRNAs, can not only be used as a signature to distinguish cancer and healthy samples, but can also serve as a predictor for overall and relapse-free survival [20,21,22]. Therefore, miRNAs have been recognized as an important class of diagnostic biomarkers for early cancer diagnosis, prognosis, and treatment [23]. Rapid and accurate measurement of miRNA expression levels is of critical importance for the evaluation of cancer risk, early detection, and the assessment of treatment efficacy.

Advances in nucleic acid-based detection have provided important tools in molecular diagnostics because of their high specificity and sensitivity. The traditional methods for miRNA detection include northern blotting, reverse-transcription quantitative PCR (RT-qPCR) and microarray [24,25,26,27]. However, these techniques require expensive reagents, sophisticated laboratory equipment and time-consuming processes that could limit the incorporation of miRNA-based diagnostics into routine clinical practice or at point-of-care. In recent years, a variety of alternative miRNA detection strategies, including isothermal amplification-based assays, electrochemical-based methods and nanomaterial-based sensing systems, have been developed [26,27,28,29,30]. However, the sensitivity and specificity of these novel techniques for miRNA detection remain challenging due to the analytical difficulties arising from the intrinsic characteristics of miRNAs, such as the short sequences, high sequence similarity and a wide range of expression levels [31,32,33].

Surface-enhanced Raman scattering (SERS) has recently attracted increasing interest for use in molecular diagnostics due to its ultra-high sensitivity and selectivity [34]. For over three decades, our laboratory has been developing a wide variety of SERS-active platforms for sensitive detection in chemical sensing and biomedical diagnostics [35,36,37,38]. Raman scattering is an inelastic scattering process where the frequency of the incident light is shifted by the vibrational energy of a molecule. This process provides unique structural fingerprints with narrow spectral bandwidths, thus allowing for selective and multiplexed detection of molecules of interest. The SERS effect occurs mainly from the electromagnetic enhancement mechanism. When shining a laser onto the surface of a metallic (such as gold or silver) nanostructure, a strong localized electromagnetic field around the nanostructure is induced by the surface plasmon, resulting in a dramatic enhancement of the Raman signal of the molecule located on or near the metallic surface. With recent advances in nanotechnology, the Raman signal of analytes can be enhanced by factors of 10^6^–10^7^, or even up to 10^12^–10^14^ at the gap (“hot spot”) between two or more adjacent nanoparticles to achieve single-molecule detection limits. For this reason, SERS is considered a powerful molecular diagnostic tool for sensitive, specific and multiplexed detection of nucleic acid biomarkers.

**Figure 1:**
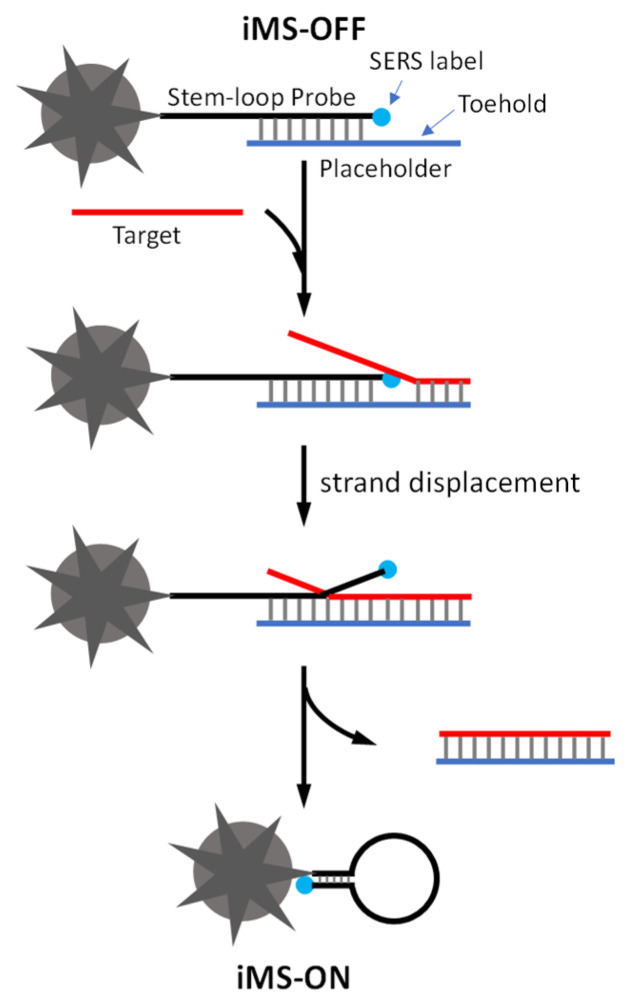
Detection scheme of the SERS iMS nanoprobe.

To detect the short miRNA sequences, we developed a sensitive and multiplexed SERS-based detection scheme referred to as the “inverse Molecular Sentinel” (iMS) [39]. The iMS sensing technique is a unique one-step homogeneous plasmonic nanobiosensor that uses a unique type of SERS-active silver-coated gold nanostars (AuNS@Ag) as the sensing platform. Gold nanostars (AuNS) developed in our laboratory can produce strong SERS at the tips of their multiple sharp branches, each with a strongly enhanced electromagnetic field. By coating them with silver, the silver-coated nanostars (AuNS@Ag) were demonstrated to offer over an order of magnitude of signal enhancement compared to uncoated AuNS. The iMS sensing mechanism is based on a non-enzymatic DNA strand-displacement process and the conformational change in stem–loop (hairpin) DNA probes for specific target identification and signal switch. As shown in Figure 1, the iMS-OFF nanoprobe is composed of a plasmonic-active nanostar (AuNS@Ag), a Raman-labeled stem–loop DNA probe and a placeholder strand. The Raman-labeled probe is functionalized on a nanostar surface via a metal–thiol bond. The placeholder strand hybridizes to the stem–loop probe keeping the Raman dye away from the nanostar surface, thus turning the SERS signal “OFF” (iMS-OFF). In the presence of targets, the miRNA target binds to the placeholder and displaces the stem–loop probe through a non-enzymatic toehold-mediated DNA strand displacement reaction. The displacement reaction is initiated by the binding of the target to the overhang region (called “toehold”) on the probe/placeholder duplex, followed by a branch migration process to displace the stem–loop probe from the placeholder. This process allows the placeholder to be released from the nanostar surface, leading to the formation of a “closed” stem–loop structure and switch the SERS signal “ON” by moving the Raman label onto the nanostar surface (iMS-ON). Using solution-based iMS assays, miR-21 has been successfully detected in total small RNA extracted from clinical esophageal samples and from breast cancer cell lines [39,40]. By using a glass SERS substrate coated with AuNS@Ag, we also demonstrated the multiplexed detection of upregulated miR-21 and miR-221, from colorectal cancer patient plasma [41].

In recent years, various signal amplification strategies have been developed for miRNA detection instead of using target amplification schemes like PCR. To achieve high sensitivity, many of the detection methods employ enzyme-assisted amplification strategies making it possible to recycle and reuse the target [42,43,44]. Generally, in these methods, the enzyme, such as Exonuclease III (Exo III), duplex-specific nuclease (DSN), or DNase I, is used to cleave or degrade only the probe strand when the probe hybridizes to its target. The target is then released to trigger a series of enzymatic cascade reactions for signal amplification. While these enzymatic strategies can effectively improve the detection sensitivity, there are still many limitations when using enzymes as they are expensive and their activities are dependent on various reaction conditions (i.e., reaction temperature, ionic strength, pH, reaction time, etc.). Thus, there has been an increasing interest in developing simple, but highly sensitive non-enzymatic (enzyme-free) signal amplification strategies for miRNA detection [27,44,45]. The most commonly used non-enzymatic signal amplification strategy is toehold-mediated strand displacement (TMSD) amplification, which utilizes the target strand as the trigger to initiate the amplification reaction [45,46]. In this strategy, the target first hybridizes to a probe strand to create an overhang as the toehold. A third strand, commonly referred to as the “fuel” strand, then binds to the probe at the toehold to initiate the strand displacement reaction, leading to release of the target that can be reused for the next amplification cycle. To date, substantial progress has been made on combining SERS and TMSD for nucleic acid detection [47,48,49,50,51,52]. However, many of the TMSD-based detection strategies still face several issues and challenges. One of the main issues is the non-specific interaction between the probe and the fuel in the absence of targets, which could increase the background signal, thus reducing the detection sensitivity. To overcome this issue, hairpin-structured DNA fuels are commonly used to prevent the non-specific interaction with the probe by hiding the complementary sequences inside the hairpin structure. However, it requires a sophisticated hairpin design to ensure the hairpin structure is sufficiently stable to prevent non-specific interaction with the probe, while not affecting the target recycling process.

The New Concept: Cascade Amplification Using the Recycling Trigger Probe (CARTP) Strategy

**Figure 2:**
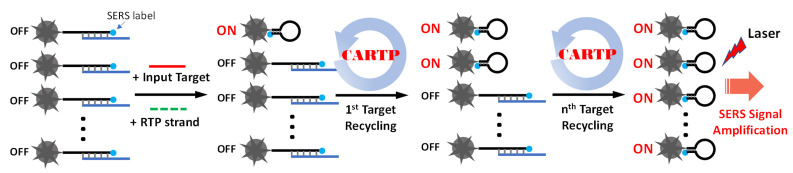
Schematic diagram showing the target recycling process for amplifying the SERS signal using the iMS nanoprobes with the CARTP strategy.

Herein, we describe a simplified non-enzymatic iMS signal amplification strategy, referred to as “Cascade Amplification by Recycling Trigger Probe” (CARTP), to improve the iMS detection sensitivity. This strategy is based on the cascade toehold-mediated DNA strand displacement reaction triggered by a “linear” DNA strand called “Recycling Trigger Probe” (RTP) strand. Figure 2 schematically shows the detection of miRNA targets using the CARTP strategy for iMS SERS signal amplification. In this strategy, the iMS-OFF nanoprobes are incubated with input targets and RTP strands. After turning on the first nanoprobe, the input target undergoes a recycling process triggered by the RTP strands. This process allows the target to turn on more iMS nanoprobes and provide an amplified SERS signal.

**Figure 3:**
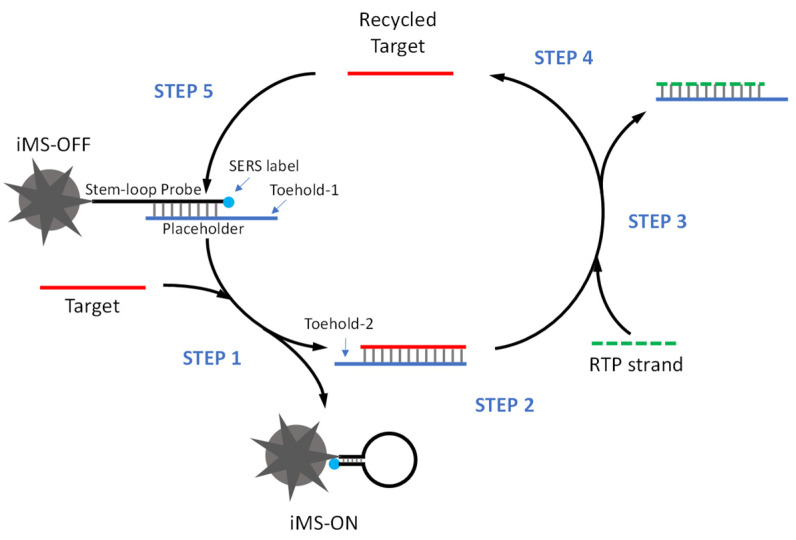
Scheme of amplified iMS sensing using the Cascade Amplification by Recycling Trigger Probe (CARTP) strategy.

Figure 3 depicts the amplified iMS detection strategy. In the presence of targets, the miRNA target binds to toehold-1 to initiate the first strand displacement reaction and turn the SERS signal “ON” (STEP 1) by releasing the target/placeholder duplex (STEP 2). The released target/placeholder duplex then serves as a substrate for the RTP strand. The “linear” RTP strand can bind to the single-stranded overhang (toehold-2) of the target/placeholder duplex to trigger the second strand displacement reaction (STEP 3), allowing the target to be released from the target/placeholder duplex (STEP 4). In this way, the released target is recycled and reused to trigger the cascade DNA strand displacement reaction (STEP 5).

**Figure 4:**
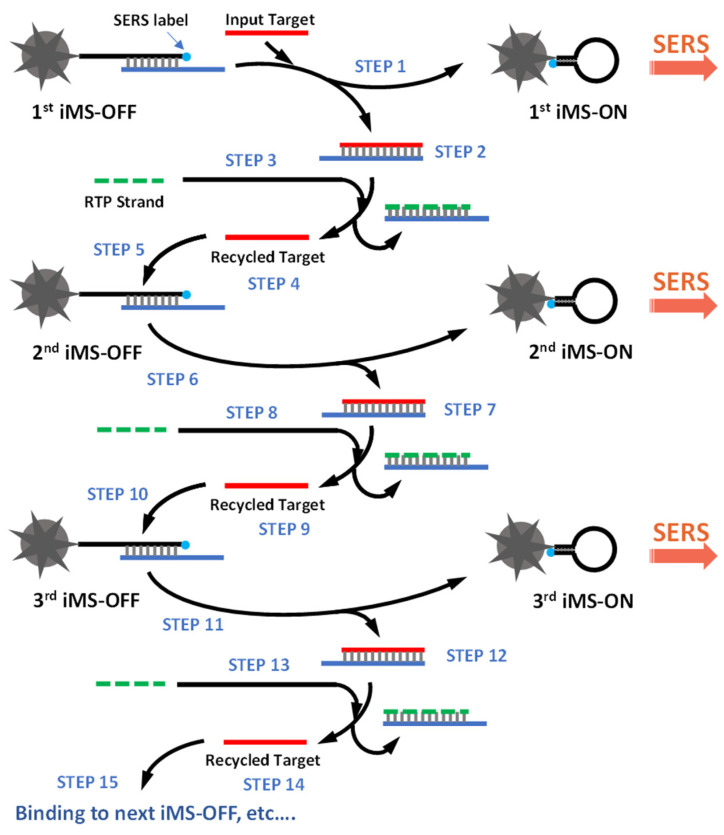
The cascade iMS amplification scheme based on the CARTP method.

Figure 4 shows the cascade amplification scheme based on the CARTP method after three cycles. After turning ON the first iMS-OFF nanoprobe, the input target is recycled at the end of each cycle (STEPS 4, 9 and 14) and subsequently turns ON more iMS-OFF nanoprobes (e.g., the second iMS-OFF, the third iMS-OFF, and so on). In this report, we demonstrate the proof-of-concept of our strategy for the first time to detect synthetic miR-21 targets with a significantly improved sensitivity and with a limit of detection (LOD) of 45 fM, which is 100-fold more sensitive than the non-amplified iMS assay used in our previous report [40].

## 2. Materials and Methods

### 2.1. Materials

Gold(III) chloride trihydrate (HAuCl_4_·3H_2_O), L(+)-ascorbic acid, silver nitrate (AgNO_3_), sodium citrate dihydrate, 1 N HCl, 6-mercapto-1-hexanol (MCH) and Thiol PEG (mPEG-SH, MW 5000) were purchased from Sigma-Aldrich (St. Louis, MO, USA). All chemicals were of the highest purity grade available and used as received. Ammonium hydroxide (NH_4_OH, 28–30%) and TCEP (Tris(2-carboxyethyl)phosphine hydrochloride) were obtained through VWR (Radnor, PA, USA). All oligonucleotides were purchased from Integrated DNA Technologies, Inc. (Coralville, IA, USA) and stored in Tris-EDTA (pH 8.0) buffer.

### 2.2. Synthesis of Silver-Coated Gold Nanostars

The silver-coated gold nanostars (AuNS@Ag) were prepared as described previously [39]. Briefly, a modified Turkevich method was used to prepare the 12 nm citrate gold nanoparticles as the gold seeds. Gold nanostars (AuNS) were then synthesized by addition of 50 μL of 6 mM AgNO_3_ to a solution containing 10 mL of 0.25 mM HAuCl_4_, 10 μL of 1 N HCl, and 100 μL of the 12 nm gold seeds under stirring at room temperature. After 5 s, 50 μL of 0.1 M ascorbic acid was added to the mixture. After stirring for 30 s, 50 μL of 0.1 M AgNO_3_ was added to the AuNS solution, followed by 10 μL of 28–30% NH_4_OH to initiate the silver coating reaction. A color change from blue to dark brown was observed in 5 min. The obtained solution was used for further functionalization with DNA probes. The stock concentration of nanostars is approximately 0.1 nM, as determined by nanoparticle tracking analysis (NTA). The characterization of the optical properties and TEM images of AuNS@Ag has been reported elsewhere [40,53].

### 2.3. Synthesis of SERS-Active iMS Nanoprobes

**Figure 5:**
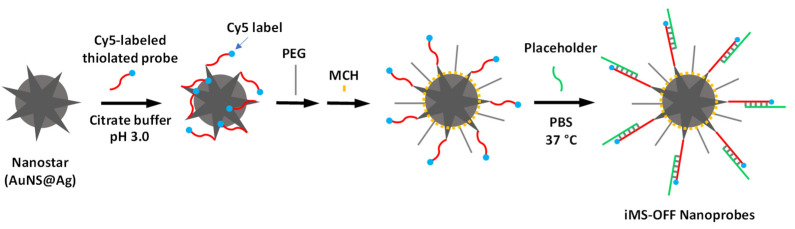
Schematic diagram illustrating the preparation process for the synthesis of the iMS nanoprobes.

The iMS nanoprobes were synthesized as described in our previous publications [39] with slight modifications according to a pH-assisted method [54]. Figure 5 shows the schematic diagram illustrating the preparation process for the synthesis of the iMS nanoprobes. To adjust the pH of the prepared AuNS@Ag solution, a citrate buffer containing 0.1 M sodium citrate dihydrate, and 0.3 N HCl was prepared. The stem–loop probes were incubated with 100× molar excess of TCEP (Tris(2-carboxyethyl)phosphine hydrochloride) at room temperature for 1.5 h to reduce disulfide bonds. The TCEP-treated probe was added to the as-prepared AuNS@Ag (1×) at a final concentration of 0.2 µM probe. The mixture (0.9 mL) was sonicated for 10 s followed by addition of 100 µL of the prepared citrate–HCl buffer. The mixture was then allowed to react at room temperature for 10 min followed by addition of 100 µL of 10 µM Thiol-PEG (mPEG-SH, MW 5000). After standing at room temperature for 30 min, the solution was mixed with 10 µL of 1% Tween-20, followed by centrifugal washing (7500× *g*, 10 min), and resuspended in 10 mM Tris HCl buffer (pH 8.0) containing 0.01% Tween-20. The nanostar surface was then passivated using 0.1 mM 6-mercapto-1-hexanol (MCH) for 10 min at 37 °C followed by four additional centrifugal washing steps (7500× *g*, 10 min) using Tris HCl buffer (10 mM, pH 8.0) containing 0.01% Tween-20. After the fourth centrifugation, the pellet was resuspended in 200 µL of 10 mM sodium phosphate buffer containing 0.01% Tween-20.

To prepare iMS-OFF nanoprobes, the iMS-AuNS@Ag solution was incubated with 2 µM placeholder DNA in 1xPBS buffer containing 0.01% Tween-20 at 37 °C for about 20 h. The excess placeholder strands were removed by four centrifugal washing steps (7500× *g*, 10 min) and finally resuspended in 1xPBS buffer containing 0.01% Tween-20. The iMS solution was then stored at 4 °C until further use.

### 2.4. iMS Assay Procedure and SERS Measurements

The iMS assay was carried out in triplicate using 5 pM iMS-OFF nanoprobes determined by nanoparticle tracking analysis (NTA 2.1, build 0342). For the CARTP assays, the miR-21 synthetic targets were first mixed with the RTP strands in a Tris-EDTA (TE) buffer. Then, aliquots of the mixture were 50 times diluted into 5 pM iMS-OFF nanoprobes in 1xPBS buffer containing 0.01% Tween-20 and 5 mM MgCl_2_ to obtain the desired concentration of the targets and 100 nM final concentration of the RTP strands. The mixture was allowed to react at room temperature for 3 or 24 h. Following the reaction, 100 µL or 5 µL of the mixture was transferred to a glass vial or a glass capillary tube, respectively, for the SERS measurements using a Renishaw InVia confocal Raman microscope equipped with a 632.8 nm HeNe laser. The light from the laser was focused into the sample solution with a 10× microscope objective after passing through a laser line filter. All SERS spectra were background subtracted and smoothed in MATLAB using a Savitsky-Golay filter with five-point window and first-order polynomial. Unless indicated otherwise, three SERS measurements were performed per sample and averaged into a single spectrum.

### 2.5. Quantification of Cy5-Labeled DNA Probes Immobilized on Silver-Coated Gold Nanostars

The number of the Cy5-labeled oligonucleotides immobilized on AuNS@Ag was determined by a dithiothreitol (DTT)-based ligand displacement assay described previously [55,56]. This DTT-based assay has been recognized as a “gold standard” for determining the surface coverage of thiolated oligonucleotides on gold nanoparticles. Briefly, 100 µL of Cy5-labeled iMS-OFF nanoprobes (final concentration 0.1 nM) was incubated with DTT (final concentration 0.5 M) in 10 mM Tris HCl buffer (pH 8.0) containing 0.01% Tween-20 for 20 h at room temperature with gentle shaking. This ligand exchange process makes it possible to displace Cy5-labeled probes from the nanostar surface completely. The solutions were centrifuged at 7500× *g* for 10 min to separate the displaced oligonucleotides from nanostars. After centrifugation, aliquots of the supernatant (50 µL) were collected and mixed with 50 µL of 10 mM Tris HCl buffer containing 0.01% Tween-20. The supernatant mixtures were then transferred into a 96-well microplate to record the fluorescence using the FLUOstar Omega microplate reader (BMG LABTECH GmbH, Ortenberg, Germany). The collected supernatants were excited at 550 nm and the fluorescent emission was measured at 580 nm. The concentrations of the released Cy5-labeled probes were determined according to a standard curve. Standard curve samples were prepared with known concentrations of the Cy5-labeled oligonucleotides using the same incubation and centrifugation procedures. The average number of oligonucleotides per nanostar was then determined by dividing the measured oligonucleotide concentration by the nanostar concentration in the sample.

## 3. Results and Discussion

### 3.1. Probe Design of CARTP Strategy for Amplification

**Figure 6:**
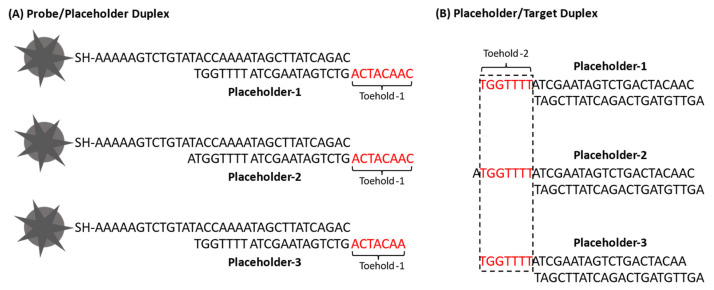
Sequence structures of (**A**) probe/placeholder duplex and (**B**) placeholder/target duplex showing the sequences of toehold-1 and toehold-2, respectively.

The cascade iMS amplification is achieved through two toehold-mediated strand displacement reactions. To initiate the amplification process, the target strand first needs to bind to the toehold-1 domain on the probe/placeholder duplex for the first strand displacement reaction. To release the target, the RTP strand needs to bind to the toehold-2 domain on the placeholder/target duplex for the second strand displacement reaction. Table 1 shows the sequences of the oligonucleotides used in this study, including the thiolated Cy5-labeled stem–loop probe, placeholders, RTP strand and target. In our previous publications [39,40], a nine-base toehold-1 domain was successfully used for the iMS assay without amplification. In this study, three placeholders were tested for the signal amplification strategy. Free energy (∆G) values of the toehold domains were also calculated using the Two-State Melting Hybridization tool on the DINA Melt web server (http://www.unafold.org, accessed on 13 July 2021). To initiate the first displacement reaction, the toehold-1 domain was designed to be eight bases with ∆G = −8.8 kcal/mol for Placeholder-1 and Placeholder-2. For Placeholder-3, the toehold-1 domain was designed to be seven bases with ∆G = −7.0 kcal/mol (Figure 6A). To trigger the second strand displacement reaction, a seven-base toehold-2 domain with a moderate binding strength (∆G = −7.0 kcal/mol) was used for all placeholders (Figure 6B) [57]. Accordingly, the stem–loop probe was modified to have two guanine (C) bases in the internal spacer instead of two adenine (A) bases in the original design from our previous publications. The Placeholder-2 was designed to have one additional base at the 3′ end to increase the stability of the probe/placeholder duplex.

### 3.2. Experimental Results

**Figure 7:**
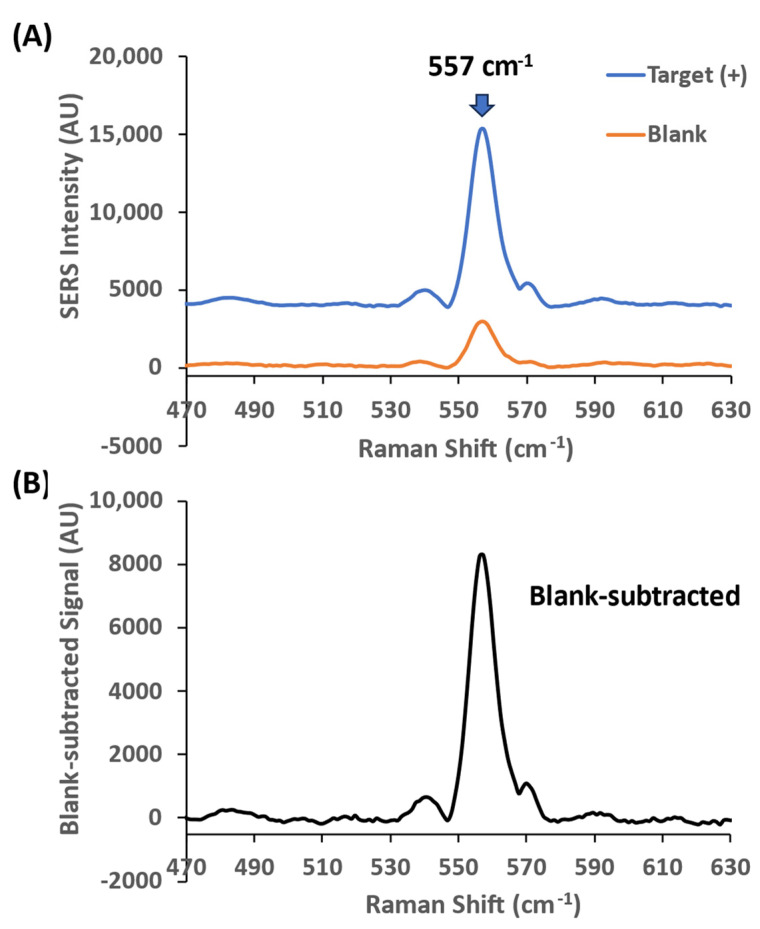
Representative SERS spectra of the iMS- nanoprobes with Placeholder-1 in the presence of 100 nM RTP strands. (**A**) The nanoprobes were incubated with 1 nM miR-21 targets (blue spectrum: Target(+)) or without targets (orange spectrum: blank) for 24 h at room temperature. The arrow indicates the 557 cm^−1^ peak of the Raman label (Cy5) conjugated on the DNA probes. (**B**) Blank-subtracted SERS signal. The spectra were taken using 4.8 mW laser power, 10 s exposure time and 5 accumulations.

To demonstrate the iMS amplification strategy, we first evaluated the amplification efficiency using the miR-21 iMS nanoprobes hybridized with three difference placeholders (Placeholder-1, Placeholder-2 and Placeholder-3). The average number of the Cy5-labeled probes immobilized on a AuNS@Ag was estimated to be 680 oligonucleotides per particle, which is 3.4 nM probes in 5 pM iMS-OFF nanostars used in this study. These iMS-OFF nanoprobes were then incubated with 1 nM synthetic DNA targets (denoted as Target(+)) in the presence (denoted as RTP(+)) or absence (denoted as RTP(−)) of 100 nM RTP strands at room temperature for 3 or 24 h. Figure 7 presents an example of representative SERS spectrum within the spectral region of the major peak of the iMS nanoprobes with Placeholder-1 in the presence of the RTP strands. The arrow indicates the main Cy5 Raman peak at 557 cm^−1^ that was used for the peak-height intensity analysis in this study. As shown in Figure 7A, the SERS intensity at 557 cm^−1^ was significantly increased after incubation with miR-21 targets, indicating that a “closed” stem–loop probe structure was formed, and the SERS signal was turned ON upon the binding of the targets. This response can be clearly seen in Figure 7B, which shows the increased SERS intensity after blank subtraction.

**Figure 8:**
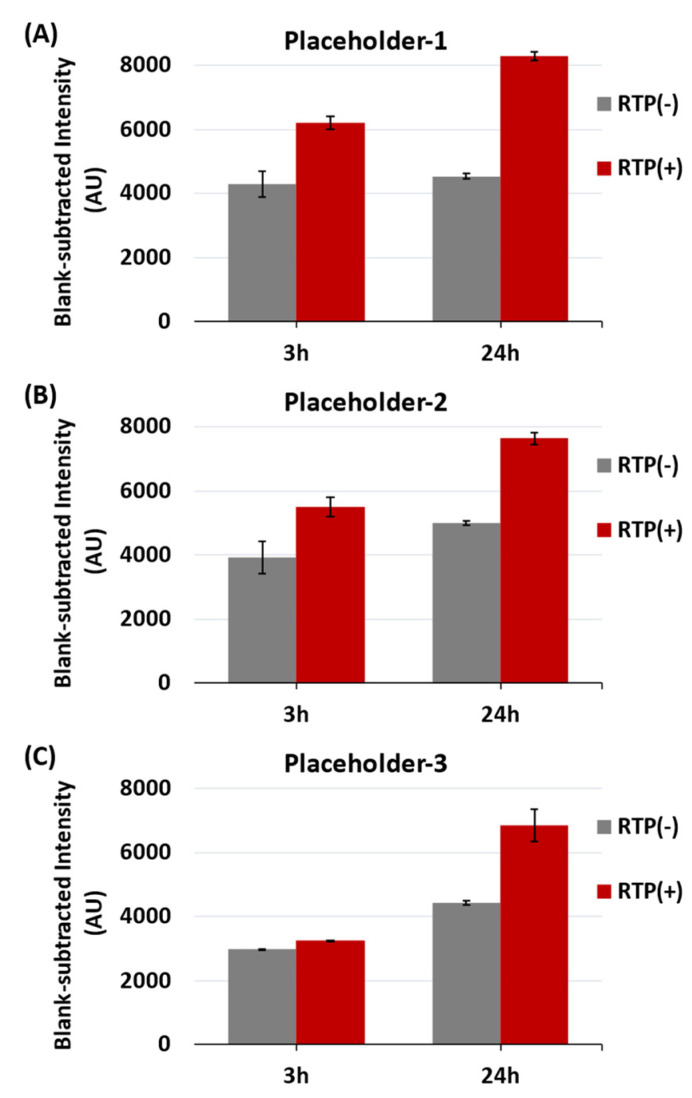
Blank-subtracted SERS peak-height intensity at 557 cm^−1^ of the miR-21 iMS nanoprobes with (**A**) Placeholder-1, (**B**) Placeholder-2, and (**C**) Placeholder-3, in the presence (denoted as RTP(+)) or absence (denoted as RTP(−)) of 100 nM RTP strands. The SERS signal was measured after 3 or 24 h incubation at room temperature with 1 nM targets (denoted as Target(+)). After incubation, 100 µL of the samples were transferred to a glass vial for SERS measurements. The spectra were taken using 4.8 mW laser power, 10 s exposure time and 5 accumulations.

In Figure 8, it can be seen that the blank-subtracted SERS peak-height intensities at 557 cm^−1^ for all three iMS-OFF nanoprobes after 24 h incubation with both targets and RTP strands (Target(+) and RTP(+)) were significantly higher than those incubated with targets but in the absence of the RTP strands (Target(+) and RTP(−)). The increased intensity (blank-subtracted intensity) was greater when Placeholder-1 was used (Figure 8A), compared to Placeholder-2 (Figure 8B) and Placeholder-3 (Figure 8C). In addition, after 3 h incubation with targets, a better amplification efficiency (i.e., a greater difference between the RTP(+) and RTP(−) samples in the presence of targets) was also found when Placeholder-1 was used, while the sample with Placeholder-3 only showed a slight difference with or without RTP strands.

The different amplification efficiencies for these three placeholders are affected by the difference in the free energy (∆G) of the toehold-1 domain; this free energy has been shown to affect the kinetics of the strand displacement [57]. The lower ∆G (−8.8 kcal/mol) observed for Placeholder-1 and Placeholder-2 demonstrates a better binding strength compared to the ∆G (−7.0 kcal/mol) for Placeholder-3. To determine the operating ionic strength for the CARTP assay, the iMS sensor response after 3 h incubation with 1 nM targets and 100 nM RTP strands in a PBS buffer containing 5 mM MgCl_2_ was compared to that in the buffer containing 2 mM MgCl_2_ (Figure S1 in Supplementary Material). A better sensor response was observed in the case of 5 mM MgCl_2_. As Placerhoder-1 and Placeholder-2 yielded the best results, they were selected for further quantification studies using the PBS buffer containing 5 mM MgCl_2_ as the reaction buffer.

To prevent or minimize the non-specific interaction between the RTP strand and probe/placeholder duplex, the RTP strand used in this proof-of-concept study does not contain the complementary sequences to the toehold-1 domain. However, an increased signal was observed for all three placeholders in the blank samples incubated only with the RTP strands (in the absence of targets), indicating the occurrence of a moderate non-specific interaction between the RTP strand and probe/placeholder duplex (Figure S2). Our results successfully demonstrated the feasibility of using the CARTP strategy for iMS signal amplification.

**Figure 9:**
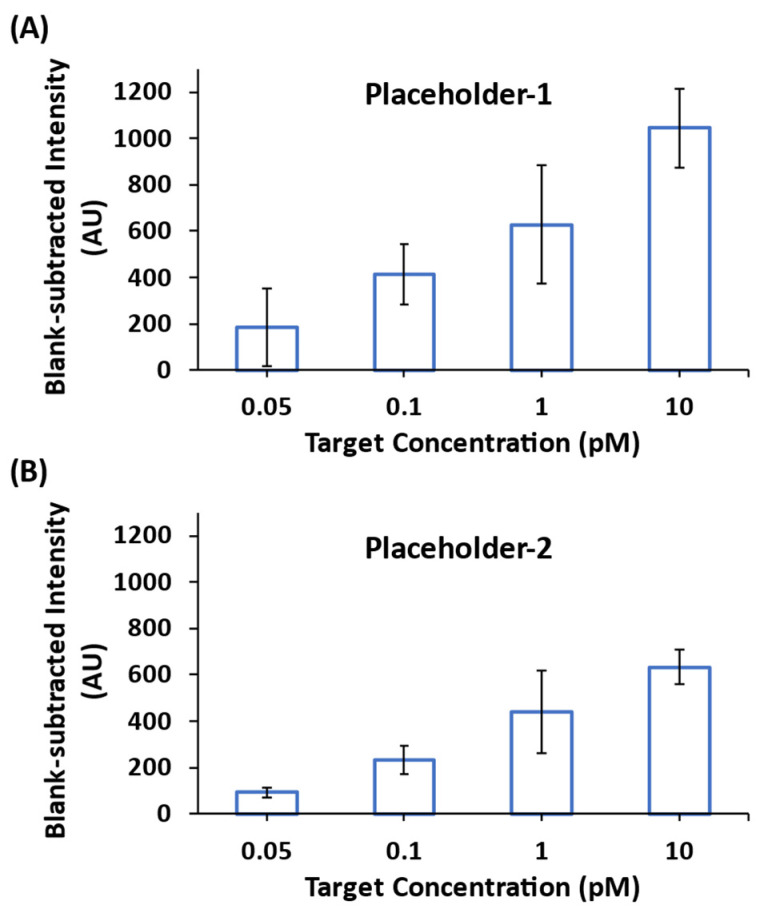
Blank-subtracted SERS peak-height intensity at 557 cm^−1^ of the miR-21 iMS nanoprobes with (**A**) Placeholder-1, and (**B**) Placeholder-2, incubated with 0.05, 0.1, 1 and 10 pM synthetic targets for 24 h at room temperature in the presence of 100 nM RTP strands for SERS signal amplification. After incubation, 5 µL of the samples were transferred to a glass capillary tube for SERS measurements. The spectra were taken using 7.3 mW laser power, 10 s exposure time and 3 accumulations.

We next investigated the detection sensitivity using the CARTP-mediated amplification strategy. The miR-21 iMS-OFF nanoprobes hybridized with either Placeholder-1 or Placerholder-2 were incubated with 0.05, 0.1, 1 and 10 pM miR-21 synthetic targets in the presence of 100 nM RTP strands for 24 h at room temperature. Figure 9 shows that the blank-subtracted SERS intensity at 557 cm^−1^ increased when increasing the target concentration from 50 fM to 10 pM for both Placeholder-1 (Figure 9A) and Placeholder-2 (Figure 9B). However, the increased SERS intensity for Placeholder-1 at each target concentration was found to be greater than that for Placeholder-2. This is caused by the one additional base at the 3′ end of Placeholder-2, which can affect the dissociation of the placeholders from the DNA probes upon target binding.

**Figure 10:**
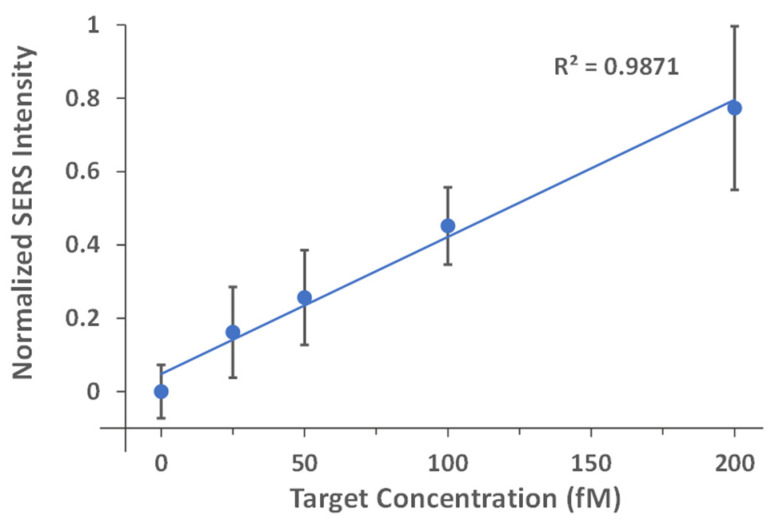
Normalized standard curves for the evaluation of the miR-21 detection sensitivity using the iMS nanoprobes with the CARTP strategy. The iMS nanoprobes with Placeholder-1 were incubated with 0 (blank), 25, 50, 100 and 200 fM synthetic targets for 24 h at room temperature in the presence of 100 nM RTP strands. After incubation, 5 µL of the samples were transferred to a glass capillary tube for SERS measurements. The spectra were taken using 7.3 mW laser power, 10 s exposure time and 3 accumulations. The SERS intensities were then blank subtracted with the average blank signal and normalized to the highest signal from the measurements.

To determine the LOD, a quantitative analysis was then performed using the iMS-OFF nanoprobes with the optimal placeholder, i.e., Placeholder-1, for target concentrations between 0 and 200 fM. The experiments were carried out in triplicate with five SERS measurements per sample on different 5 µL aliquots to minimize the variance between experiments. As shown in Figure 10, a linear trend line was fitted to the data (normalized blank-subtracted SERS intensity at 557 cm^−1^) for target concentrations at 0, 25, 50, 100, and 200 fM with R^2^ value of 0.9871. The LOD was then determined to be 45 fM based on the 3σ-rule by using the best-fit linear equation and the standard deviation of the normalized intensity from the blank. This result shows that the CARTP amplification strategy provides a significantly improved detection sensitivity, which is 100-fold more sensitive than the non-amplified iMS assay used in our previous work [40].

## 4. Conclusions

In conclusion, we demonstrated, for the first time, the feasibility of the CARTP strategy for cascade iMS amplification catalyzed by a “linear” RTP strand. We show that our simplified iMS amplification assay with a LOD of 45 fM provides a 100-fold improved sensitivity compared with the non-amplified iMS assay in our previous work. Further optimization of the approach will be performed in future studies in order to address various aspects. For example, various RTP strand configurations could be further investigated in order to minimize the possible non-specifically interaction with the iMS-OFF nanoprobe or the interaction between the recycled target and the toehold-1 domain on the placeholder/RTP duplex. To address these aspects, future studies will focus on designing and optimizing the RTP strands and assay conditions, including, but not limited to, the RTP length or different configurations, reaction buffer, assay time, reaction temperature, etc. The probe/placeholder loading capacity and the concentrations of the RTP strands and nanoprobes will be further optimized to increase the dynamic range. Additionally, placeholders with a range of binding strength for the toehold-1 and toehold-2 domains will be systematically investigated for different target sequences in future studies.

In recent years, several alternative miRNA sensing strategies have been developed in order to facilitate the translation of miRNA biomarkers from basic research to clinical application. For example, when combining SERS and DNA strand displacement amplification, miRNA detection with LODs ranging from sub-fM to sub-pM can be achieved in 30 min to several hours [47,48,49,50,51]. These SERS signal amplification assays provide wide dynamic ranges spanning five to eight orders of magnitude. However, many of them require a sophisticated design of hairpin structured strands to trigger the amplification process. Additionally, some of these methods require multiple washing/rinsing steps or magnetic separation. In other approaches, anti-DNA/RNA antibodies have been used for the detection of miRNAs using other sensing methods, including Reflective Phantom Interface (RPI) technology, surface plasmon resonance imaging (SPRi) and enzyme-based amperometric sensing [58,59,60]. These antibody-based assays generally provide LODs at sub-pM levels with dynamic ranges spanning three to four orders of magnitude. However, like enzymes, antibodies are more expensive and are susceptible to various reaction and storage conditions compared to DNA. Moreover, these substrate-based assays require either rinsing steps or fabrication of different sensing spots to detect multiple targets.

In contrast, the iMS sensing technology developed in our laboratory is a unique homogeneous bioassay, which does not require PCR, target labeling or any subsequent washing steps. Our method can be used in both solution-based and substrate-based assays for various applications. The multiplexed capability of the iMS sensing based on SERS also offers significant advantages over other optical methods, such as fluorescence and chemiluminescence. Multiplexed detection can be easily achieved in a one-pot format as multiple targets can be detected in a single solution or in the same spot on a SERS substrate by using different Raman labels. It is worth noting that our method can be performed using a small sample volume (a few µL) in a capillary tube that could be advantageous for clinical applications when limited quantities of samples can be collected. We anticipate that further optimization of the CARTP assay will reduce the assay time, increase the dynamic range, and improve the LOD for advanced applications, such as Point-of-Care (POC) testing.

In addition to focusing on assay optimization, future work will also involve using this iMS amplification strategy for multiplexed detection of miRNAs from clinical specimens. Our previous pilot studies successfully demonstrated the use of the iMS technique without the amplification strategy to detect miR-21 from clinical esophageal samples [40] and miR-21 and miR-221 from colorectal cancer patient plasma [41]. Future studies will expand this work to include multiple miRNA biomarkers and large numbers of actual clinical samples for improved diagnostic accuracy and capability; also, comparison of iMS data with RT-qPCR results will help validate the reliability and robustness of this CARTP-based iMS assay. We envision that this method will ultimately allow detection of multiple miRNA biomarkers with ultra-high sensitivity for future clinical translation and application.

## Figures and Tables

**Table 1:** Oligonucleotide sequences used in this study.

Name	Sequence (5′ → 3′)
Stem–loop probe *	thiol-AAAAAGTCTGTATACCAAAATAGCTTATCAGAC-Cy5
Placeholder-1 **	CAACATCAGTCTGATAAGCTATTTTGGT
Placeholder-2 **	CAACATCAGTCTGATAAGCTATTTTGGTA
Placeholder-3 **	AACATCAGTCTGATAAGCTATTTTGGT
RTP strand	ACCAAAATAGCTTATCAGAC
Target	TAGCTTATCAGACTGATGTTGA

* Sequences in red represent the modified bases compared to the original design in previous publications [39,40]. ** Underlined sequences represent the toehold-1 domain for the target strand at the 5′ end and the toehold-2 domain for the RTP strand at the 3′ end.

## Data Availability

Data are contained within the article or supplementary material.

## Supplementary Materials

The following supporting information can be downloaded at: https://www.mdpi.com/article/10.3390/bios13080774/s1, Figure S1: Blank-subtracted SERS spectra of the miR-21 iMS nanoprobes with (A) Placeholder-1, (B) Placeholder-2, and (C) Placeholder-3, in the presence 100 nM RTP strands and 1 nM targets (denoted as Target(+) & RTP(+)). The reactions were performed in 1xPBS buffer containing either 5 mM (blue spectrum) or 2 mM MgCl_2_ (red spectrum) for 3 hours at room temperature. After incubation, 100 µL of the samples were transferred to a glass vial for SERS measurements. The spectra were taken using 4.8 mW laser power, 10 s exposure time and 5 accumulations.; Figure S2: SERS peak-height intensity at 557 cm^−1^ of the miR-21 iMS nanoprobes with (A) Placeholder-1, (B) Placeholder-2, and (C) Placeholder-3, in the presence (denoted as RTP(+)) or absence (denoted as RTP(-)) of 100 nM RTP strands. The SERS signal was measured after 3- or 24-hour incubation at room temperature with 1 nM targets (denoted as Target(+)) or without targets (denoted as Blank). After incubation, 100 µL of the samples were transferred to a glass vial for SERS measurements. The spectra were taken using 4.8 mW laser power, 10 s exposure time and 5 accumulations.
