# Supplementary material for: Cascade Amplified Plasmonics Molecular Biosensor for Sensitive Detection of Disease Biomarkers
Source: Biosensors (Basel). 2023 Jul 31;13(8):774. doi: 10.3390/bios13080774 (PMC10452163; doi:10.3390/bios13080774)
Supplement: Supplementary file 1 [file biosensors-13-00774-s001.zip › biosensors-2481641-supplementary.pdf]

biosensors

Supplementary Information

Cascade Amplified Plasmonics Molecular Biosensor for Sensitive Detection of Disease Biomarkers

Hsin-Neng Wang [1,2] and Tuan Vo-Dinh [1,2,3,*]

1 Fitzpatrick Institute for Photonics, Duke University, Durham, NC 27708, USA; hsinneng.wang@duke.edu
2 Department of Biomedical Engineering, Duke University, Durham, NC 27708, USA
3 Department of Chemistry, Duke University, Durham, NC 27708, USA
* Correspondence: tuan.vodinh@duke.edu

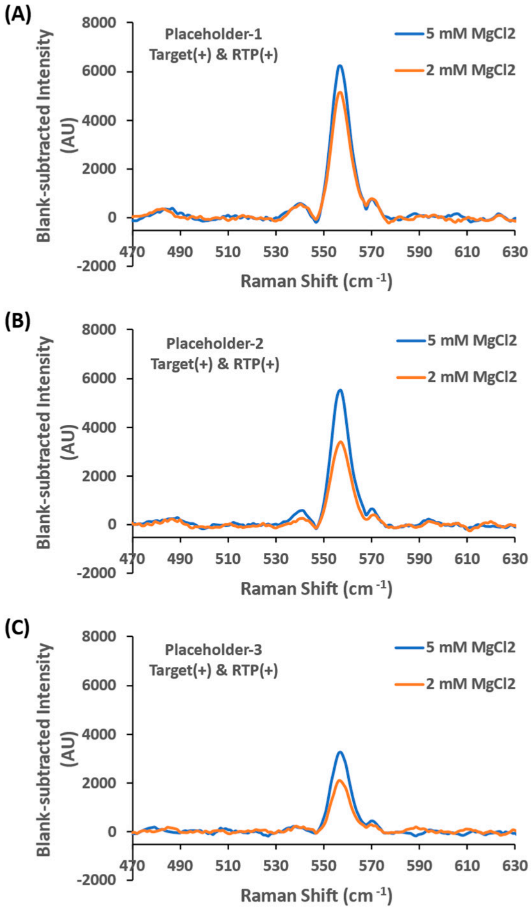

Figure S1. Blank-subtracted SERS spectra of the miR-21 iMS nanoprobes with (A) Placeholder-1, (B) Placeholder-2, and (C) Placeholder-3, in the presence 100 nM RTP strands and 1 nM targets (denoted as Target(+) & RTP(+)). The reactions were performed in 1xPBS buffer containing either 5 mM (blue spectrum) or 2 mM $MgCl_2$ (red spectrum) for 3 hours at room temperature. After incubation, 100 µL of the samples were transferred to a glass vial for SERS measurements. The spectra were taken using 4.8 mW laser power, 10 second exposure time and 5 accumulations.

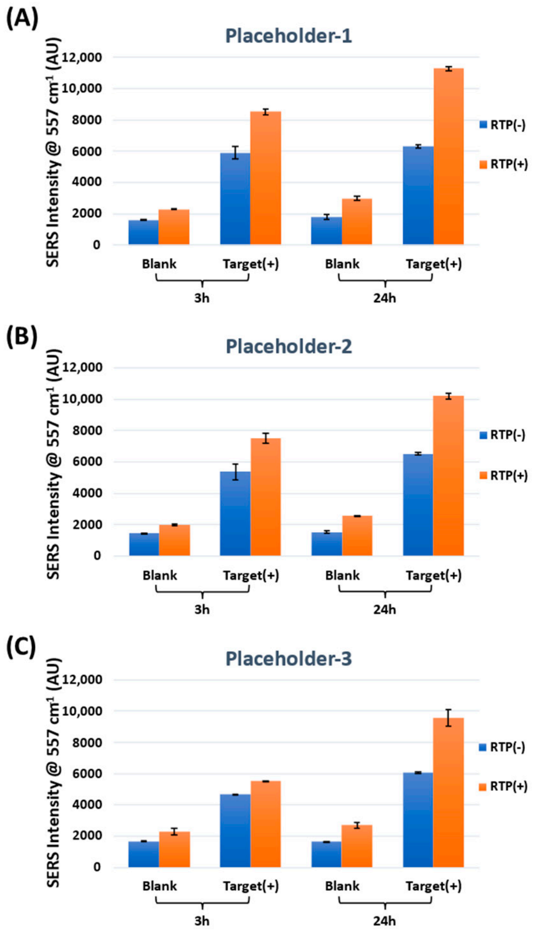

Figure S2. SERS peak-height intensity at 557 cm^{-1} of the miR-21 iMS nanoprobes with (A) Placeholder-1, (B) Placeholder-2, and (C) Placeholder-3, in the presence (denoted as RTP(+)) or absence (denoted as RTP(-)) of 100 nM RTP strands. The SERS signal was measured after 3- or 24-hour incubation at room temperature with 1 nM targets (denoted as Target(+)) or without targets (denoted as Blank). After incubation, 100 μL of the samples were transferred to a glass vial for SERS measurements. The spectra were taken using 4.8 mW laser power, 10 second exposure time and 5 accumulations.
